# Antifungal Nail Lacquer for Enhanced Transungual Delivery of Econazole Nitrate

**DOI:** 10.3390/pharmaceutics14102204

**Published:** 2022-10-16

**Authors:** Vinam Puri, Riya Savla, Kevin Chen, Keyaara Robinson, Amitkumar Virani, Bozena Michniak-Kohn

**Affiliations:** 1 Department of Pharmaceutics, Ernest Mario School of Pharmacy, Rutgers, The State University of New Jersey, Piscataway, NJ 08855, USA; 2 Center for Dermal Research, Life Science Building, Rutgers, The State University of New Jersey, Piscataway, NJ 08854, USA; 3 Department of Cell Biology and Neuroscience, Rutgers, The State University of New Jersey, Piscataway, NJ 08855, USA

**Keywords:** onychomycosis, ungual drug delivery, topical antifungal treatment, econazole nitrate, nail formulation, quality by design, lacquer characterization, drying test, handwashing simulation

## Abstract

The fungal disease of the nail, onychomycosis, which is also the most prevalent nail disturbance, demands effective topical treatment options considering the possible adverse effects of systemic antifungal therapy. The current work is focused on development of an adhesive and resistant, drug-delivering and permeation-enhancing polymeric film containing econazole nitrate (ECN) for topical antifungal treatment. The development of the lacquer formulation was guided by the Quality by Design approach to achieve the critical quality attributes needed to obtain the product of desired quality. Eudragit RSPO at 10% *w*/*w* was found to be the ideal adhesive polymer for the application and an optimal permeation-enhancing lacquer formulation was achieved by the optimization of other formulation excipients, such as plasticizer and the solvent system. Additionally, novel experimental enhancements introduced to the research included refined D_50_ drying time and drying rate tests for lacquer characterization as well as a multi-mechanism permeation-enhancing pre-treatment. Moreover, a practical implication was provided by a handwashing simulation designed to test the performance of the lacquer during actual use. In vitro drug release testing and ex vivo nail permeation testing demonstrated that the optimized nail lacquer performed better than control lacquer lacking the permeation enhancer by achieving a faster and sustained delivery of ECN. It can be concluded that this is a promising drug delivery system for topical antifungal treatment of onychomycotic nails, and the novel characterization techniques may be adapted for similar formulations in the future.

## 1. Introduction

The fungal disease of the nail, onychomycosis is the single most prevalent disturbance of the nail. It is characterized by discoloration, brittleness and thickening of the infected nail and is caused predominantly by dermatophytes but also by non-dermatophyte molds and yeasts [1]. Aside from the unpleasant appearance of the affected nail, certain severe cases of the disease can also be painful, with the pain even becoming debilitating at times [2]. There is also a link seen between onychomycosis and other fungal infections such as tinea pedis, tinea manuum, etc., and it is believed that infections of the nail could spread to other parts of the body or vice versa [3,4]. There is also a link between onychomycosis and HIV, and the disease could be a signal for immunocompromise in patients [5,6,7]. It could also be a marker of diabetes mellitus and lack of glycemic control, which is also a risk factor for the disease [8,9]. Other conditions that might be risk factors for onychomycosis are trauma [10], age [11], and peripheral vascular disease [12], while genetics also plays a role [13]. Moreover, there have also been studies suggesting nail fungus could cause an impact on the psychological and social wellbeing of patients and could affect their self-confidence as well as quality of life, even hindering their ability to go to work [14,15]. With a rising prevalence worldwide and higher prevalence in older patients or those with comorbidities, this is an important disorder that needs to be addressed. 

Currently approved approaches for the treatment of onychomycosis include systemic therapy by means of oral treatment or topical application of antifungal formulations [16,17]. There is low preference of oral treatment seen with patients and physicians because of the known potential adverse side effects, especially hepatic [18,19]. Topical formulations do not carry a high enough efficacy and are limited by the impermeability of the nail plate to hydrophobic antifungal drugs [16,20]. Topical treatment often needs to be combined with oral antifungals to achieve the desired effects [21,22]. However, there are constant efforts being made by researchers to make antifungals available topically to the site of infection, and some have shown some promise as well [23,24]. The recent approaches to achieve favorable transungual drug delivery of antifungals include applications of modified delivery systems such as colloidal particles [25,26] or chemical or mechanical methods of penetration enhancement with application of topical formulations [27,28,29]. Chemical methods of permeation enhancement have certain advantages over physical methods, namely, being cheaper, incorporating into the formulation or application with the formulation, as well as their ease of use, which enables application without trained professionals [30].

The structure of the nail plate mostly consists of a tightly packed network of keratin fibers along with a low content of lipids (0.1–1%) and water (10–25%) [27,31,32]. The nail plate has been referred to as a hydrogel by many scientists and it has been seen that a better hydrated nail plate is more permeable because of loosened structured and increased porosity [33,34,35,36]. This is thought to occur by increased flexibility and segmental mobility of the keratin matrix upon hydration by means of modification of Van der Waals forces, hydrogen bonding and ionic interactions between the matrix and proteins [35,36]. 

From a formulation point of view, solutions and lacquers have been most popular, although creams and gels have also been tried as vehicles for drug delivery. Lacquers are a promising delivery system because they are based on the idea that upon evaporation of the solvent, they form polymeric drug-loaded films on the tissue which have a high concentration of the drug and enable a prolonged contact with the tissue [37]. This provides the concentration gradient of the drug needed for passive diffusion to occur through the nail plate. They are also very resistant to environmental degradation from the daily activities performed by the patient. There is also a concern, however, that the film formed might be too dehydrated and may not allow the drug to be released from the polymer matrix [38]. Chemical penetration enhancement has been one of the more widely used formulation techniques to help with drug permeation. There have been several chemical penetration enhancers incorporated into lacquers to help with penetration improvement. Another approach for penetration enhancement is dipping the nail in a pretreatment solution, such as papain, prior to treatment with the formulation. 

According to the ICH guidelines Q8 (R2), Q9 and Q10, the development of products should be performed with a science-based and risk-managed approach called Quality by Design (QbD) [39,40,41]. This approach involves defining a quality target product profile (QTPP) and identifying the critical quality attributes (CQAs) of the product [42]. Finally, based on the understanding of the process for development of the product, the critical material attributes (CMAs) as well as critical process parameters (CPPs) are identified, and experiments are designed to provide a statistically supported range for these variables that would allow a quality product to be developed [42].

Econazole is an imidazole antifungal drug which has been shown to have a broad spectrum against a variety of fungal organisms and also against some bacteria, owing to its ability to permeate the cells of these pathogens [43]; in addition, it is applied in its nitrate form for the treatment of several fungal conditions of the skin and has been investigated for transungual delivery as well [44]. It causes its antifungal effect by inhibition of 14α-lanosterol demethylase, which is an important enzyme in the ergosterol synthesis, leading to membrane disruption of the fungal cells [45]. 

Our research is based on the learnings from topical formulation for ungual drug delivery that a hydrated nail with a compromised keratin network will allow higher permeation of econazole nitrate (ECN) through the nail plate from an occlusive and resistant lacquer formulation. In the current work, we have formulated a polymeric nail lacquer containing econazole nitrate which was evaluated for the drug release and potential for permeation enhancement along with several permeation enhancers. Additionally, a multi-mechanism permeation-enhancing pre-treatment was used prior to the application of the lacquer, which consisted of a solution of humectant and a keratolytic agent providing enhanced drug permeation by two simultaneous mechanisms. We found that the permeation of econazole into the nail plate was higher with the test antifungal lacquer following pretreatment as compared to a control antifungal lacquer without the selected permeation enhancer, following pretreatment. Also presented are some novel methods for characterization of antifungal lacquers to support improved statistical analysis as well as practical relevance of the product. This is a promising drug delivery system to attain desirable levels of antifungal drugs following topical application.

## 2. Materials and Methods

### 2.1. Materials

Econazole Nitrate (≥98%) was purchased from Cayman Chemical Company (Ann Arbor, Michigan, USA). Eudragit polymers and Hydroxypropyl Methylcellulose (HPMC) were kind gifts from Evonik Corporation (Piscataway, New Jersey, USA) and Ashland (Covington, Kentucky), respectively. Transcutol P was a gift from Gattefosse (Paramus, New Jersey, USA). Kolliphor CS 20 and Kolliphor RH 40 were generous gifts from BASF (Tarrytown, New York, USA). Isopropyl Alcohol (IPA), Acetone, Ethanol (200 proof), HPLC grade water, Tween 80, Terpineol, Polyethylene Glycol 400 and Salicylic Acid were purchased from Sigma-Aldrich, Inc. (St. Louis, Missouri, USA). Triacetin was purchased form TCI Chemicals (Portland, Oregon, USA), Papain was purchased from MP Biomedicals, LLC (Irvine, California, USA) and phosphate-buffered saline (PBS) was purchased in the form of tablets from Tocris, Bio-Techne Corporation (Minneapolis, Minnesota, USA). Dexpanthenol was purchased from Spectrum Chemical Mfg. Corp. (New Brunswick, New Jersey, USA), Urea was purchased from Santa Cruz Biotechnology (Dallas, TX). Human cadaver fingernail samples were purchased from Science Care (Phoenix, Arizona, USA). (Note: Cadaver tissue samples were obtained from an accredited Tissue Bank and could not be linked to specific individuals and, therefore, did not require approval from an Ethics Committee or Institutional Review Board.)

### 2.2. Preparation of the Antifungal Nail Lacquer

The antifungal lacquer was prepared by first dissolving the film forming polymer in an adequate solvent using stirring, with or without heat, depending on the polymer. After complete dissolution of the polymer, the plasticizer was added to the solution while stirring. Then, the drug solubilizer was added with continuous stirring, followed by the active ingredient, ECN. Finally, the permeation enhancer was added to the solution while stirring and allowed to dissolve to complete the formulation.

### 2.3. Selection and Optimization of Excipients

The selection of excipients for the preparation of the nail lacquer and the optimization of their amounts was performed using the QbD approach. The QTPP was defined in tabular along with the justification of the target choice of each element. This quality was determined based on prior knowledge about antifungal topical nail products and literature review of antifungal nail lacquers. Screening of the excipients was then performed using design of experiments (DoE) function of JMP^®^ statistical software by SAS. Randomized experimental trials were conducted with different combinations of material variables, and the results were analyzed for statistical impact on product quality. 

Some pilot experiments were conducted for the evaluation of a hybrid film-forming polymer system consisting of a hydrophilic polymer, HPMC E100 with a hydrophobic polymer, Eudragit RL100 (E-RL100), dissolved in a hydroalcoholic solvent system containing ethanol and water. Other excipients in this pilot study included PEG 400 as a plasticizer and humectant, and 2-mercaptoethanol as a permeation enhancer. The polymer solutions were prepared by dividing the polymers in relevant solvents and mixing the two solutions. The total polymer content was varied while keeping the ratio of the two polymers fixed at 70:30 (E-RL100/HPMC) based on previously published literature [46]. The composition of the formulations made for the preliminary screening of excipients have been summarized in Table 1. 

For the finalization of the type of polymer, solvent system, as well as plasticizer for the lacquer was performed with a secondary DoE which screened excipient combinations out of four polymers, two solvent systems and three plasticizers, as shown in Table 2. These trials consisted of fixed amounts of these ingredients while only their types were varied. The order of experiments and combinations in each experiment was randomly determined by JMP^®^ Pro 15 (SAS Institute Inc., Cary, North Caroline, USA).

The final round of DoE consisted of experimental trials with varied amounts of the selected polymer and plasticizer combination, based on the data generated from the secondary DoE. Partial factorial designs were generated using JMP^®^ Pro 15 (SAS Institute Inc., Cary, North Caroline, USA) and it was also utilized to perform the statistical analysis of the responses generated from these trials. The parameters in the formulations that were kept fixed are also presented in Table 3, along with the low (-1), middle (0) and high (+1) levels of the polymer and plasticizer. 

Selection of the permeation enhancer was performed after the rest of the composition of the lacquer was finalized and maximal drug loading was achieved. The screened permeation enhancers were added to the nail lacquer at the concentration of 5% *w*/*w* and were allowed to stir under magnetic stirring at 500 RPM overnight in tightly closed scintillation vials. Aqueous solutions of the permeation enhancers at the same concentrations were also made using the same process. The permeation enhancers used for this selection were: dexpanthenol, urea, diethylene glycol monoethyl ether (Transcutol P), terpineol, polyoxyl 20 cetostearyl ether (CS 20), polyoxyl 40 hydrogenated castor oil (RH 40), PEG 400, and salicylic acid. The lacquers with permeation enhancers were subjected to hydration efficiency [28,47,48,49] and drug uptake enhancement testing [50]. Hydration efficiency factor (HEF) represented the change in the weight of the nail upon soaking a pre-weighed piece of nail overnight into the aqueous solution of the permeation enhancer as a ratio of change in nail weight upon soaking a pre-weighed piece of the nail plate in water over the same time as that of the permeation enhancer solutions. Drug uptake enhancement factor (UEF) represented as the ratio of the amount of drug uptake into the nail after soaking a pre-weighed piece of the nail plate in the lacquer formulation containing the permeation enhancer to the amount of drug uptake in the nail plate upon soaking in the lacquer without permeation enhancer. Equations (1) and (2) show the formulae for calculation of the HEF and UEF, respectively.

(1) $$HEF=\frac{Increase\;in\;nail\;weight\;in\;PE\;solution}{Increase\;in\;nail\;weight\;in\;water}$$

where HEF is hydration efficiency factor and PE is permeation enhancer.

(2) $$UEF=\frac{Amt\;of\;drug\;uptake\;\left(per\;unit\;nail\;wt\right)\;with\;drug+enhancer\;formulation}{Amt\;of\;drug\;uptake\;\left(per\;unit\;nail\;wt\right)\;with\;drug\;only\;formulation}$$

where UEF is drug uptake enhancement factor.

### 2.4. Characterization of the Antifungal Nail Lacquers

#### 2.4.1. Drying Time Test

The drying time test used for preliminary lacquer formulations was a standard test that has been extensively used for nail lacquers in the literature. Briefly, a brush applicator was used to spread the lacquer on a 2 cm^2^ area marked on a glass slide. A gloved finger was used to touch the film every 15 s, and the time taken to achieve a dry-to-the-touch film was recorded.

When testing the lacquers from the final optimization DoE, a novel, more sensitive method for measuring relative drying times was used to effectively see the difference in drying times between lacquers and to obtain statistically meaningful information from the generated response. A 10 µL aliquot of each lacquer was pipetted into a weigh boat, and the initial stable weight was recorded. The weigh boat was left on the weighing scale as the solvents started evaporating and the lacquer started drying. With an accurate stopwatch, the time taken for the lacquer to drop to half of the initial weight was recorded as the *D*_50_ *drying time*. This measurement could be reported as a function of the initial lacquer weight representing the *D*_50_ *drying rate* as shown in Equation (3). All measurements were performed in triplicate, and means were reported for the statistical analysis of the responses.

(3) $$D_{50}\;drying\;rate=\frac{Initial\;weight\;of\;the\;lacquer}{D_{50}\;drying\;time\;of\;the\;lacquer}$$

where *D*_50_ *drying time* is the time taken for the initial weight of 10 µL of the lacquer to drop by 50%.

#### 2.4.2. Water Resistance Test

The lacquer was applied with a brush applicator within a rectangular area of 2 cm × 3 cm on a pre-weighed glass slide and allowed to dry. Weight of the completely dried film was measured, and the slides were immersed vertically in deionized water for 24 h, making sure that the film was completely under water. After 24 h, the slides were removed and were patted dry using lint-free wipes to be weighed again. The loss in weight of the lacquer as a percentage of the original film weight was reported as a measure of its water resistance as shown in Equation (4) [51,52].

(4) $$Water\;resistance=\frac{\left(Initial\;film\;weight-final\;film\;weight\right)}{Initial\;film\;weight}\times 100$$

#### 2.4.3. Adhesion Test

Adhesion testing was performed on the lacquers using previously published methodology [51,53]. Using a pipette, a 10 µL drop of the lacquer was applied on glass slide and allowed to dry completely. Care was taken to maintain the same distance between the pipette tip and the glass slide during repeated applications to obtain the same spread of the drop. Upon drying, the film formed on the slide was cut into squares with a knife. The number of squares formed on each dried drop of lacquer was kept constant to ensure comparable analysis. Then, a piece of tape was applied on the films with constant pressure and stripped with one quick motion. The number of squares of the film that were remaining on the slide after tape stripping were reported as a percentage of the total number of squares as a measure of adhesion property of the lacquer.

#### 2.4.4. Viscosity

The viscosity of the optimized lacquers was measured using a Brookfield RVT Viscometer (Brookfield Engineering Laboratories, Middleboro, MA, USA) with Spindle #2 at 50 rpm at 25 °C. 

### 2.5. Handwashing Simulation Study

This simulation study was conducted by measuring and estimated amount of hand soap and water utilized for each handwash performed for an average individual. The lacquer was applied on a glass slide with a brush applicator, and the weight of the dried film was recorded. Then, the slide with the dried lacquer film was inserted into a beaker containing the simulated handwash soap solution (0.12 % *v*/*v* liquid hand soap), and the solution was stirred with magnetic stirring at 500 rpm for 20 s. Following this, the slide was removed from the beaker and pat dry with lint-free wipes and re-weighed. The weight of the lacquer films after three rounds of simulated handwashing was recorded.

### 2.6. In Vitro Drug Release Testing

The in vitro release testing (IVRT) of ECN from the antifungal nail lacquers was profiled using SnakeSkin^TM^ dialysis tubing, 10K MWCO (Thermo Fisher Scientific, Waltham, MA, USA) which was sandwiched between the donor and receptor chambers of vertical Franz diffusion cells (Logan Instruments, Somerset, New Jersey, USA) with a receptor volume of 5 mL and an effective diffusion area of 0.64 cm2. The receptor compartment containing a 3 mm magnetic stir bar was filled with PBS (pH 7.4) containing 30% (*v*/*v*) ethanol to achieve sink conditions. The assembled Franz cells were kept in FDC-24 heat blocks (Logan Instruments, Somerset, New Jersey, USA) set at 37°C, which stirred the receptor media at 600 rpm for 15 min for equilibration before dosing with 100 µL of the lacquer formulations containing 5% *w*/*w* of different permeation enhancers in the donor compartment. The control formulation for this study was the ECN nail lacquer without any permeation enhancer. Then, 300 µL samples were withdrawn through the sampling arm of the Franz cells at predetermined time points followed by immediate replenishment with the same volume of fresh receptor media. The pre-validated HPLC method was used to determine the drug content in each sample, and cumulative drug contents were plotted against the time to obtain the release profile.

### 2.7. Ex Vivo Nail Permeation Study

Frozen human cadaver fingernail samples were obtained from Science Care, Phoenix, AZ and were stored at −20 °C before use. Nail plates from index, middle and ring fingers of the donors were used because of their structural similarity. Nail plates were thawed at room temperature (25 ± 2 °C) and rinsed with DI water, cleaned by removal of adhering tissues with forceps, followed by another rinse with DPBS. The cleaned nails were then briefly immersed in 70% ethanol for disinfection, then dried with lint-free wipes. Cleaned nails were visually inspected for any cracks or fractures and characterized in terms of weight and thickness to be selected for the permeation study. The selected nail plates were free from any visual cracks, were of similar weights and were of 0.7 mm ± 0.15 mm in average thickness, measured at three different points using a digital micrometer (Mitutoyo, Kawasaki, Kanagawa, Japan). A permeation enhancement pre-treatment of the nail plates was performed as a modification of previously demonstrated treatments [54,55]. An aqueous solution of 10% *w*/*v* TGA and 10% *w*/*v* PEG 400 was used for the pre-treatment of the nails achieved by overnight incubation of the nail plates. After removal of the nails from the pre-treatment solution, they were dried and utilized for permeation testing. 

Modified vertical Franz diffusion cells clamped with Neoflon^®^ nail adapters (PermeGear, Hellertown, Pennsylvania, USA) having an effective exchange area of 0.2 cm^2^ were used for the nail permeation experiment. The receptor chamber consisted of 5 mL of DPBS (pH 7.4) with 30% *v*/*v* of ethanol, maintained at 37 °C and stirred constantly at 600 rpm. Then, 200 µL of the test and control lacquers were applied to the exposed portion of the dorsal surface of the nail plates mounted on the adapters. Then, 300 µL samples were collected from the sampling arm of the Franz cells at 0 h, 12 h, 24 h, 36 h and 48 h time points followed by daily sampling for up to 7 days, and an equal volume of fresh receptor media was replenished after every sampling.

### 2.8. Analytical Method for Drug Determination

The content of ECN in samples from the various tests was quantified using a high-performance liquid chromatography (HPLC) system (Agilent 1100 Series) with autosampler coupled with a UV detector using a reverse phase C-18 column (150 mm X 4.6 mm; 5 µm) at room temperature. The mobile phase consisted of an isocratic elution using an 85:15 mix of methanol and water as the mobile phase with a run time of 10 min at a flow rate of 1 mL/min. The UV detection was performed at 230 nm, and the analyte peak was observed at around 4.5 min. The method was validated with respect to linearity, precision, accuracy, and repeatability with a calibration standard range of 0.1 µg/mL to 500 µg/mL with a coefficient of correlation, R2 >0.99. The intra-day and inter-day coefficient of variance ranged from 0.11% to 1.33% and 0.22% to 1.91%, respectively, and accuracy was found to range from 98.22% to 100.21% and 98.29% to 101.5%, respectively.

### 2.9. Statistical Analysis

All measurements were performed with 3–5 replicates, as described under respective sections and standard deviations were calculated. Statistical comparison between groups was performed using an unpaired *t* test where applicable, with *p* ≤ 0.05 considered as the threshold for significance. Randomized experimental trial designs and statistical analysis on experimental data was performed using JMP^®^ Pro 15 (SAS Institute, Cary, NC, USA).

## 3. Results and Discussions

Application of the QbD approach for development of the lacquer allowed a systematic way of selection of formulation variables to lead to the final optimized lacquer product. In a recent study utilizing the QbD approach, liposomes of an antifungal drug were delivered using a lacquer formulation [48]. QbD was applied for optimization of the liposomes, and the lacquer formulation was formulated with a fixed formula, and standard characterization of lacquer was performed along with permeation experiments with the product [48]. Another recent study focused on developing an itraconazole nail lacquer optimized by central composite design based design of experiments for the selection of polymer content (4% *w*/*v* to 6% *w*/*v*) and permeation enhancer (2.5% *v*/*v* to 5% *v*/*v*) [52].

### 3.1. QTPP and Excipient Selection

Based on the QTPP for the antifungal nail lacquer, as shown in Table 4, there were three CQAs that were identified to guide the development of the product—drying time, water resistance and adhesion. A lacquer that leaves a dry-to-the-touch film relatively quickly, does not get washed off easily with day-to-day activities and adheres well to the nail while meeting the other QTPP elements will be a desirable product which would favor relatively higher efficiency and patient compliance. The lacquers obtained from the DoE trials were evaluated for these three characteristics before screening the optimized formulation for further qualities.

The pilot experiments were performed to screen whether a combination of polymers in a hydroalcoholic solvent system would be suitable for the purposes of our research as such a system has been reported previously in the literature, stating beneficial properties of the resultant lacquer, such as capability to hydrate the nail with the hydrophilic component while achieving desirable physical characteristics of the lacquer such as resistance and adhesion with the hydrophobic component [51]. Even bilayered systems have been reported to have similar benefits with a hydrophilic and hydrophobic coat of the lacquers applied separately [56]. With the preliminary screening experiments, we did not observe the desired qualities in the lacquers as all the lacquers showed a drying time of longer than 2 min. Moreover, it was observed that none of the films obtained with such a combination system were completely dry to the touch at any point during testing. Therefore, from the observations of the preliminary DoE, the secondary design prepared contained no hydrophilic component, and organic solvent systems reported previously to result in lacquers with desirable properties were used further [28,53]. 

From the secondary trials, it was observed that the IPA:Butanol solvent system was not able to completely dissolve the formulation excipients and none of the trials with IPA:Butanol resulted in a clear solution as the product. The concentrations at which the polymer was used in the formulations was not suitable for this solvent system. Therefore, IPA:Acetone was used as the solvent to conduct the remaining trials in the design and the responses obtained from the trials have been compiled in Table 5.

The results from this experimental trial showed that all lacquers containing triacetin as the plasticizer displayed a 100% adhesion property while the water resistance for all lacquers was not statistically different. As for the drying time response, it was observed that the combination of Triacetin with all polymers but E-RSPO showed drying times over 2 min. This led to the selection of the E-RSPO as the polymer and triacetin as the plasticizer for further optimization.

The next step was to finalize the amount of each of these excipients to result in lacquers of desirable properties, which was achieved by means of the final DoE, the results from which are presented in Table 6. We believe that the *D_50_ drying time* methodology developed in our work is a significant improvement in the currently used method and can provide a much-improved way for product developers to statistically access the quality of their product. Up until recently, similar products have been tested by an outdated approach which may not be as accurate when comparing formulations [48,52]. When testing drying times by touching the films to see if they are dry, there can be a gap of several seconds between touches and the time actual time by which the lacquer film dries may be missed, making it less accurate, especially when several formulations are being compared with drying time as a critical quality attribute.

It was found from the analysis of the responses that the concentrations of the plasticizer did not significantly impact the product CQAs that were measured as the responses. Therefore, it was decided to reduce the usage of the excipient to the minimum value as that still resulted in desirable lacquer characteristics, so triacetin concentration was optimized to 10 % *w*/*w*. However, in case of the polymer, that decision was not taken due to the drying time response. There appeared to be a direct relationship between the polymer concentration and drying time of the lacquer. The 30 % *w*/*w* concentration of the polymer that showed drying times greater than 2 min was therefore removed from further analysis. Another factor impacting the choice of polymer concentration was the content of the drug that could be incorporated into the polymer matrix. Lacquers with 10 % (LQ10) and 20 % *w*/*w* (LQ20) polymer concentrations were made, and the maximum drug that could be loaded into these systems was tested. Table 7 shows the composition.

Higher concentrations than 2 %*w*/*w* and 2.25 % *w*/*w* in LQ10 and LQ 20, respectively, did not retain the drug in solubilized form, leaving these combinations as the final ones. Since both the drug loads were not very different, further characterization was performed on both lacquers. 

**Figure 1:**
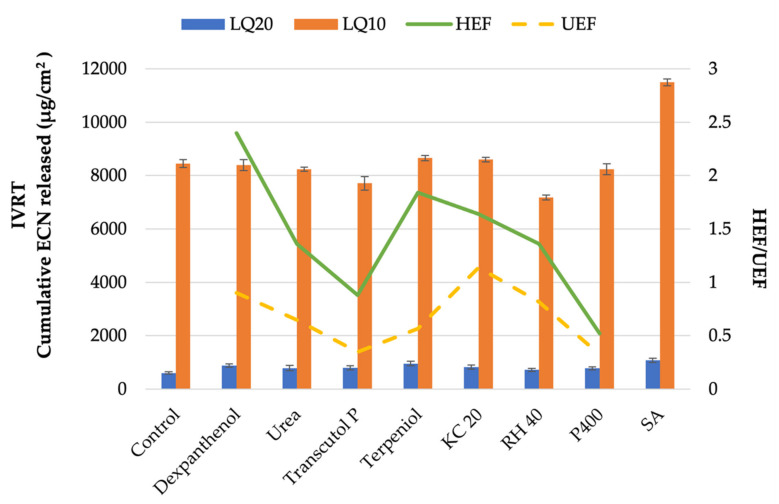
Comparative analysis of lacquers containing 10% *w*/*w* polymer (LQ10) and 20 % *w*/*w* polymer (LQ20) with different permeation enhancers, showing cumulative amount of econazole nitrate released on the primary Y-axis, and hydration efficiency factor (HEF) and drug uptake enhancement factor (UEF) on the secondary Y-axis. The bars represent the cumulative drug release from in vitro release tests (IVRT), solid and broken lines represent the HEF and UEF values, respectively.

For the selection of the permeation enhancers, the lacquers were made with different permeation enhancers to test them for hydration efficiency and drug uptake enhancement. Additionally, in vitro release testing was performed with these lacquers as well, and the results from these studies have been presented together in Figure 1.

From the analysis of the drug release, HEF and UEF, KC 20 was selected as the permeation enhancer for the optimized formulation. Dexpanthenol showed the highest HEF value, but drug UEF was highest for KC 20, and similar drug release was observed with both enhancers. The HEF/UEF tests were excluded for SA, since it was not soluble at the concentration the enhancers were being added. In terms of drug release, it was observed in general that LQ10 released much higher amounts of ECN as compared to LQ20, despite the amount of drug loaded in the latter being higher. This may be explained by the fact that the lacquer with the higher polymer concentration had more available spaces in the matrix for the drug particles as it formed the film, making it harder for the drug particles to diffuse out of the polymer matrix, whereas the lower polymer content lacquer had a more saturated polymer matrix, making it easier for the drug to be released. This finding was consistent with similar studies in the literature that observed a reduction in drug release with an increased concentration of polymers [28,53].

### 3.2. Characterization of the Antifungal Nail Lacquers

The optimized nail lacquer formulation and a control lacquer (without permeation enhancer), compositions of which are shown in Table 8, were further characterized as described below.

The viscosity of the test and control lacquers were found to be a 50.7 ± 4.6 cP and 41.3 ± 6.1 cP, respectively, consistent with similar products made previously [51]. This provides the lacquer with the desirable consistency to spread easily on the nail and form the fast-drying film.

**Figure 2:**
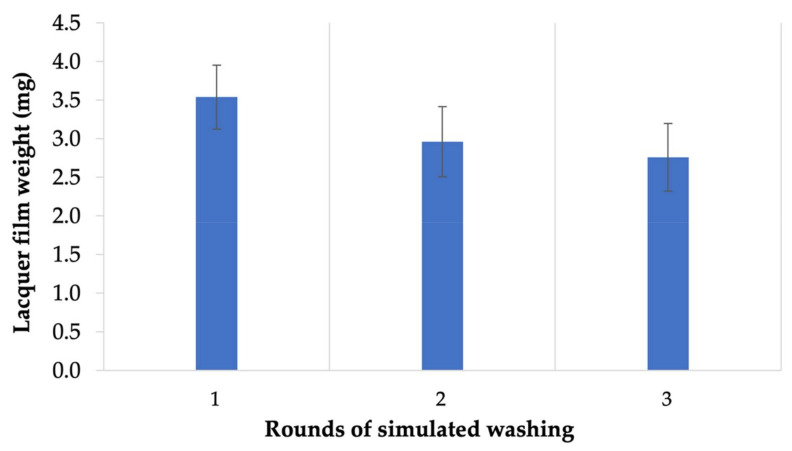
Mean lacquer film weights after each round of simulated handwashing ± SD (n = 5), a novel approach to realistically test nail lacquers for patient use.

The handwashing simulation study was a new way developed by us to demonstrate the practical applicability of the nail lacquer. Since the nail lacquers applied to fingernails will have a significant exposure to water with the day-to-day activities performed, we developed a simulation to show how well the lacquer could be expected to stay on the nails with such activity. Three rounds of simulated handwashing were used with the optimized nail lacquer for this analysis, and the responses obtained are displayed in Figure 2. From the simulation, it was seen that the lacquer obtained was a meaningful product for practical application as the weight of the film formed on the glass surface did not change significantly.

It is also being highlighted that the new drying time designed for the purposes of a more sensitive testing of nail lacquers was an important addition to the generally followed practices for lacquer characterization. Since the traditional method of drying time testing involves touching the film at defined intervals to record the time when it is dry to the touch, it is possible to miss the exact time the lacquer takes to dry as it may be happening in between the intervals at any time. The *D*_50_ *drying time* test developed during this research is a more accurate method, especially when comparing products that may present very similar drying times. The *D*_50_ *drying rate* is another way to represent the rate at which the lacquer dries. The traditional technique may be used for initial screening and to ensure the product characteristic is in the desired range and this newer method could be used for closely comparing within that range. We believe these drying time and rate metrics could provide much more meaningful analysis, especially during final selection of similar lacquer products and could allow statistical comparisons when it may not be possible with the traditional testing methodology.

### 3.3. In Vitro Drug Release Testing

**Figure 3:**
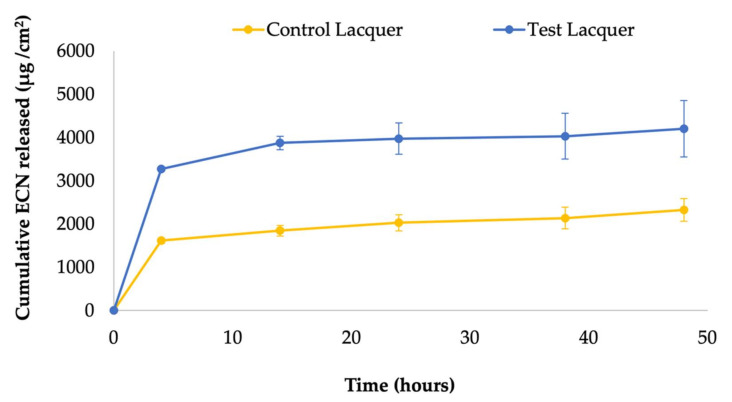
In vitro drug release studies showing cumulative amount of econazole nitrate released from the test and control lacquers across a unit area of the permeation membrane over time, reported as mean ± SD; n = 4 for test lacquer and n = 3 for control lacquer.

The IVRT of the control and test lacquer showed significant difference in the release of the drug from the two lacquers. It was seen that test lacquer showed a higher amount drug released from the polymer film as compared to the control lacquer throughout the 48 h study. The comparison of the release from the two lacquers is represented in the plot shown in Figure 3.

This demonstrates that the presence of the permeation enhancer is also an important contributor in allowing the drug to diffuse out from the polymer matrix.

### 3.4. Ex Vivo Nail Permeation Testing

The nail permeation study performed with human cadaver nails using modified Franz cells was the final analysis performed to ensure that the lacquer can deliver the drug into the nail unit. 

The pretreatment step performed was a modification and likely enhancement of the currently reported pretreatment procedures in the literature that either use one type of permeation for the treatment or follow a sequential approach to pretreatment [55,57]. This practice of pretreatment is supposed to be a solution for a situation where the permeation enhancer is incompatible with the nail formulation but can also be used to achieve higher efficiency of drug delivery. Our pretreatment approach was as multi-mechanism approach where the two permeation enhancers in the pretreatment solution act on the nail by different mechanisms. The solution used consisted of thioglycolic acid which is a keratolytic agent that acts by breaking the disulfide linkages holding the keratin units together in the nail plate. The other component of the solution was PEG 400 which enhances permeation into the nail plate by the mechanism of hydrating it and causing it to swell, which results in the formation of channels in the nail plate, allowing higher drug permeation. The combination approach, therefore, is expected to be an improvement on existing approaches. 

**Figure 4:**
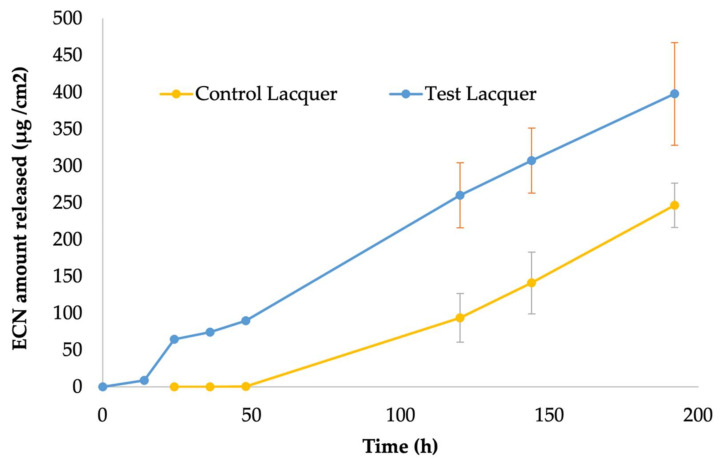
Ex vivo nail permeation study showing cumulative amount of econazole nitrate permeated though a unit area of human cadaver nails from topically applied test and control nail lacquers, reported as mean ± SD; n = 5 for test lacquer, and n = 3 for control lacquer.

The results of nail permeation of ECN are presented In Figure 4. It was seen that even with the pretreatment step, the presence of KC 20 in the test lacquer made it favorable over the control lacquer, as detectable amounts of the drug were seen in the receptor medium within the first 24 h of permeation, whereas the control did not show drug permeation even on day 2 of the IVPT. Over the 7 days of the study, the test lacquer maintained higher accumulation of drug through the nail plate and appears to be a favorable formulation for transungual drug delivery.

## 5. Conclusions

The QbD approach was utilized to screen through excipients over multiple rounds of DoE to obtain an optimized antifungal nail lacquer formulation for the topical delivery of econazole nitrate. Our research also presented some new methods for more sensitive and practical testing of lacquer characteristics. The *D*_50_ *drying time* test and *D*_50_ *drying rate* were novel ways to report the drying time and could provide a mor accurate way to compare lacquers with similar performance when traditional drying time testing methodology may not provide statistically sensitive data. Additionally, the handwashing simulation was another innovation in the characterization of nail lacquer products to provide evidence of practicality of this drug delivery system. A topical nail delivery product that would be able to sustain effect of daily washing activities would carry the potential to deliver the antifungal more effectively and could also improve patient compliance when there is less restriction on the patients for daily activities. Finally, it can be concluded that the developed lacquer system is a promising delivery system for antifungal drugs following topical application, and the optimization approach could be used to identify different combinations for a variety of antifungal drugs.

## Figures and Tables

**Table 1:** Preliminary lacquer formulations showing different combinations of the excipients.

Formulation #	1	2	3	4	5	6	7	8	9	10	11	12	13	14	15
**ECN (%*w*/*w*)**	1	3	5	7	10	1	3	5	7	10	1	3	5	7	10
**Ethanol/Water**	80:20					70:30									
**Polymer Content (%*w*/*w*)**	5										7.5				
**2-mercaptoethanol (%*w*/*w*)**	5														
**PEG 400 (%*w*/*w*)**	10														

ECN: Econazole nitrate.

**Table 2:** Levels of chosen material attributes (X1, X2 and X3) and responses measured (Y1, Y2 and Y3) as well as parameters fixed for the secondary experimental design.

Factors (Independent Variables)			Responses (Dependent Variables)	Fixed Parameters
X1	X2	X3		
Polymer	Solvent	Plasticizer		
E-RL100	IPA:Acetone	Glycerol	Y1 Drying Time	Batch Volume—10 mL
E-RLPO	IPA:Butanol	PEG 400	Y2 Water Resistance	Polymer Content—30% *w*/*w*
E-RS100		Triacetin	Y3 Adhesion	Plasticizer Concentration—30% *w*/*w*
E-RSPO				Drug Solubilizer (Tween 80)—0.25% *w*/*w*
				IPA:Butanol—5:1
				IPA:Acetone—3:2

E-RL100: Eudragit RL 100; E-RLPO: Eudradit RLPO; E-RS100: Eudragit RS 100; E-RSPO: Eudragit RSPO.

**Table 3:** Levels of chosen material attributes (X1 and X2) and responses measured (Y1, Y2 and Y3) as well as parameters fixed for the final experimental design.

Factors (Independent Variables)			Responses (Dependent Variables)	Fixed Parameters
	X1 (%*w*/*w*)	X2 (%*w*/*w*)		
Levels	E-RSPO Concentration	Triacetin Concentration		
−1	10	10	Y1 D_50_ Drying Time	Batch Volume—10 mL
0	20	20	Y2 Water Resistance	Drug Solubilizer Tween 80—0.25% *w*/*w*
+1	30	30	Y3 Adhesion	IPA:Acetone—3:2

E-RSPO: Eudragit RSPO; levels of independent variables −1, 0, +1 represent low, medium and high values of the factors varied over the conducted experiments.

**Table 4:** Quality target product profile (QTPP) for antifungal nail lacquer.

QTPP Element	Target	CQA	Justification
Dosage form	Nail lacquer		Promising transungual dosage form as demonstrated in the literature
Route of administration	Topical		To prevent adverse drug effects and delivery at the site of infection
Dosage strength	Maximal drug-loading		To achieve drug load > MIC: 0.2 µg/mL
Drying time	<2 min	✓	To prevent removal while drying and to increase patient compliance
Water resistance	At least 24 h	✓	To prevent washout with daily handwashing
Adhesion	Maximize	✓	To prevent removal of drug film with daily activities

CQA: critical quality attributes; MIC: minimum inhibitory concentration.

**Table 5:** Randomized experimental trials for the antifungal lacquer formulations with two controlled variables and three dependent responses for the selection of the types of polymer and plasticizer.

Factors (Independent Variables)		Responses (Dependent Variables) *		
X1	X2	Y1 (s)	Y2 (%)	Y3 (%)
Polymer	Plasticizer	Drying Time	Water Resistance	Adhesion
RLPO	Triacetin	165	33.8	100
RSPO	Glycerol	45	32.6	0
RLPO	Glycerol	45	32.4	77.78
RS100	PEG 400	105	30.1	0
RSPO	PEG 400	90	29.6	88.89
RSPO	Triacetin	105	28.9	100
RL100	PEG 400	150	34.4	100
RL100	Glycerol	75	33.8	11.11
RS100	Glycerol	60	31.7	100
RL100	Triacetin	165	33.7	100
RS100	Triacetin	135	33.2	100
RLPO	PEG 400	120	31.9	100

* All responses recorded are mean values (n = 3). RLPO: Eudragit RLPO; RSPO: Eudragit RSPO; RS100: Eudragit RS 100; RL100: Eudragit RL 100

**Table 6:** Randomized experimental trials for the antifungal lacquer formulations containing one polymer and one plasticizer, at three levels each, and resulting responses.

Factors (Independent Variables)		Responses (Dependent Variables) *		
X1 (% *w*/*w*)	X1 (% *w*/*w*)	Y1 (s)	Y2 (%)	Y3 (%)
RSPO Concentration	Triacetin Concentration	D_50_ Drying Time	Water Resistance	Adhesion
10	20	39	28	100
10	30	45	38	100
20	20	87.33	16	100
30	30	168	30	100
20	30	84.33	28	100
10	10	49	18	100
20	10	89.5	12	100
30	20	207	25	100
30	10	120.67	27	100

* All responses recorded are mean values (n=3). RSPO: Eudragit RSPO.

**Table 7:** Final lacquer comparison to check maximum drug loading.

Excipient	Concentration (% *w*/*w*)	
	LQ10	LQ20
E-RSPO	10	20
Triacetin	10	10
ECN	2	2.25

E-RSPO: Eudragit RSPO; ECN: econazole nitrate.

**Table 8:** Composition of the final optimized test and control lacquers.

Excipient	Concentration (% *w*/*w*)	
	Test Lacquer	Control Lacquer
E-RSPO	10	10
Triacetin	10	10
ECN	2	2
Kolliphor CS 20	5	-
Tween 80	0.25	0.25
Solvent	QS to 100	QS to 100

E-RSPO: Eudragit RSPO; QS: quantum satis (sufficient quantity to make up the volume).

## Data Availability

Not applicable.

## References

[1] To M.J., Brothers T.D., Van Zoost C. (2016). Foot Conditions among Homeless Persons: A Systematic Review. PLoS ONE.

[2] Drake L.A., Scher R.K., Smith E.B., Faich G.A., Smith S.L., Hong J.J., Stiller M.J. (1998). Effect of onychomycosis on quality of life. J. Am. Acad. Dermatol..

[3] Szepietowski J.C., Reich A., Garlowska E., Kulig M., Baran E., Group f.t.O.E.S. (2006). Factors Influencing Coexistence of Toenail Onychomycosis With Tinea Pedis and Other Dermatomycoses: A Survey of 2761 Patients. Arch. Dermatol..

[4] Sigurgeirsson B., Steingrímsson Ó. (2004). Risk factors associated with onychomycosis. J. Eur. Acad. Dermatol. Venereol..

[5] Gupta A.K., Taborda P., Taborda V., Gilmour J., Rachlis A., Salit I., Gupta M.A., MacDonald P., Cooper E.A., Summerbell R.C. (2000). Epidemiology and prevalence of onychomycosis in HIV-positive individuals. Int. J. Dermatol..

[6] Gupta A.K., Daigle D., Foley K.A. (2015). The prevalence of culture-confirmed toenail onychomycosis in at-risk patient populations. J. Eur. Acad. Dermatol. Venereol..

[7] Jing W., Ismail R. (1999). Mucocutaneous manifestations of HIV infection: A retrospective analysis of 145 cases in a Chinese population in Malaysia. Int. J. Dermatol..

[8] Akkus G., Evran M., Gungor D., Karakas M., Sert M., Tetiker T. (2016). Tinea pedis and onychomycosis frequency in diabetes mellitus patients and diabetic foot ulcers. A cross sectional - observational study. Pak. J. Med. Sci..

[9] Vlahovic T.C., Sebag J.A., Tosti A., Vlahovic T.C., Arenas R. (2017). Onychomycosis in Diabetics. Onychomycosis: An Illustrated Guide to Diagnosis and Treatment.

[10] Avner S., Nir N., Henri T. (2006). Fifth toenail clinical response to systemic antifungal therapy is not a marker of successful therapy for other toenails with onychomycosis. J. Eur. Acad. Dermatol. Venereol..

[11] Araiza-Santibáñez J., Tirado-Sánchez A., González-Rodríguez A.L., Vázquez-Escorcia L., Ponce-Olivera R.M., Bonifaz A. (2016). Onychomycosis in the elderly. A 2-year retrospective study of 138 cases. Rev. Med. Hosp. Gen..

[12] Bang C.H., Yoon J.W., Lee H.J., Lee J.Y., Park Y.M., Lee S.J., Lee J.H. (2018). Evaluation of relationships between onychomycosis and vascular diseases using sequential pattern mining. Sci. Rep..

[13] Lipner S.R., Scher R.K. (2019). Onychomycosis: Clinical overview and diagnosis. J. Am. Acad. Dermatol..

[14] Kayarkatte M.N., Singal A., Pandhi D. (2020). Impact of Onychomycosis on the Quality of Life: Dermatology Life Quality Index-Based Cross-Sectional Study. Skin Appendage Disord..

[15] Gupta A.K., Mays R.R. (2018). The Impact of Onychomycosis on Quality of Life: A Systematic Review of the Available Literature. Skin Appendage Disord..

[16] Lipner S.R., Scher R.K. (2019). Onychomycosis: Treatment and prevention of recurrence. J. Am. Acad. Dermatol..

[17] Roberts D.T., Taylor W.D., Boyle J. (2003). Guidelines for treatment of onychomycosis. Br. J. Dermatol..

[18] Gupta A.K., Paquet M. (2015). Management of Onychomycosis in Canada in 2014. J. Cutan. Med. Surg..

[19] Austin John Maddy A.T., Rigopoulos D., Richert B. (2018). Systemic Treatment of Onychomycosis. Onychomycosis: Diagnosis and Effective Management.

[20] Scher R.K. (1996). Onychomycosis: A significant medical disorder. J. Am. Acad. Dermatol..

[21] Olafsson J.H., Sigurgeirsson B., Baran R. (2003). Combination therapy for onychomycosis. Br. J. Dermatol..

[22] Shemer A., Gupta A.K., Babaev M., Barzilai A., Farhi R., Daniel Iii C.R. (2018). A Retrospective Study Comparing K101 Nail Solution as a Monotherapy and in Combination with Oral Terbinafine or Itraconazole for the Treatment of Toenail Onychomycosis. Skin Appendage Disord..

[23] Gupta A., Joseph W. (2000). Ciclopirox 8% nail lacquer in the treatment of onychomycosis of the toenails in the United States. J. Am. Podiatr. Med. Assoc..

[24] Bunyaratavej S., Leeyaphan C., Rujitharanawong C., Surawan T.M., Muanprasat C., Matthapan L. (2016). Efficacy of 5% amorolfine nail lacquer in Neoscytalidium dimidiatum onychomycosis. J. Dermatol. Treat..

[25] Flores F.C., Rosso R.S., Cruz L., Beck R.C.R., Silva C.B. (2017). An innovative polysaccharide nanobased nail formulation for improvement of onychomycosis treatment. Eur. J. Pharm. Sci..

[26] Bseiso E.A., Nasr M., Sammour O.A., Abd El Gawad N.A. (2016). Novel nail penetration enhancer containing vesicles “nPEVs” for treatment of onychomycosis. Drug Deliv..

[27] Angelo T., Borgheti-Cardoso L.N., Gelfuso G.M., Taveira S.F., Gratieri T. (2017). Chemical and physical strategies in onychomycosis topical treatment: A review. Med. mycol..

[28] Khattab A., Shalaby S. (2018). Optimized Ciclopirox-Based Eudragit RLPO Nail Lacquer: Effect of Endopeptidase Enzyme as Permeation Enhancer on Transungual Drug Delivery and Efficiency Against Onychomycosis. AAPS PharmSci. Tech..

[29] Lee B.C., Pangeni R., Na J., Koo K.-T., Park J.W. (2019). Preparation and in vivo evaluation of a highly skin- and nail-permeable efinaconazole topical formulation for enhanced treatment of onychomycosis. Drug Deliv..

[30] Murdan S. (2008). Enhancing the nail permeability of topically applied drugs. Expert Opin. Drug Deliv..

[31] Walters K.A., Abdalghafor H.M., Lane M.E. (2012). The human nail – Barrier characterisation and permeation enhancement. Int. J. Pharm..

[32] Elsayed M.M.A. (2015). Development of topical therapeutics for management of onychomycosis and other nail disorders: A pharmaceutical perspective. J. Control. Release.

[33] Murdan S. (2002). Drug delivery to the nail following topical application. Int. J. Pharm..

[34] WALTERS K.A., FLYNN G.L. (1983). Permeability characteristics of the human nail plate. Int J. Cosmet. Sci..

[35] Gunt H.B., Kasting G.B. (2007). Effect of hydration on the permeation of ketoconazole through human nail plate in vitro. Eur. J. Pharm. Sci..

[36] Benzeval I., Bowen C.R., Guy R.H., Delgado-Charro M.B. (2013). Effects of Iontophoresis, Hydration, and Permeation Enhancers on Human Nail Plate: Infrared and Impedance Spectroscopy Assessment. Pharm. Res..

[37] Tabara K., Szewczyk A.E., Bienias W., Wojciechowska A., Pastuszka M., Oszukowska M., Kaszuba A. (2015). Amorolfine vs. ciclopirox - lacquers for the treatment of onychomycosis. Adv. Dermatol. Allergol..

[38] Murdan S. (2014). Focal drug delivery to the nail. Focal Controlled Drug Delivery.

[39] ICH Q8 (R2) (2009) Pharmaceutical Development. https://www.ich.org/fileadmin/Public_Web_Site/ICH_Products/Guidelines/Quality/Q8_R1/Step4/Q8_R2_Guideline.pdf.

[40] ICH Q9 (2005) Quality Risk Management. https://www.ich.org/fileadmin/Public_Web_Site/ICH_Products/Guidelines/Quality/Q9/Step4/Q9_Guideline.pdf.

[41] ICH Q10 (2008) Pharmaceutical Quality System. https://www.ich.org/fileadmin/Public_Web_Site/ICH_Products/Guidelines/Quality/Q10/Step4/Q10_Guideline.pdf.

[42] Schlindwein W.S., Gibson M. (2018). Pharmaceutical Quality by Design: A Practical Approach.

[43] Srivastava S., Verma U., Kumar R., Bhatt N. (2021). Preparation and Evaluation of Econazole Nitrate Containing Film-Forming Gel. Eur. J. Mol. Clin. Med..

[44] Aggarwal R., Targhotra M., Sahoo P., Chauhan M.K. (2020). Onychomycosis: Novel strategies for treatment. J. Drug Deliv. Sci. Technol..

[45] Sharma R., Pathak K. (2011). Polymeric nanosponges as an alternative carrier for improved retention of econazole nitrate onto the skin through topical hydrogel formulation. Pharm. Dev. Technol..

[46] Patel M.M., Vora Z.M. (2016). Formulation development and optimization of transungual drug delivery system of terbinafine hydrochloride for the treatment of onychomycosis. Drug Deliv. Transl. Res..

[47] Alqahtani A., Raut B., Khan S., Mohamed J.M.M., Fatease A.A., Alqahtani T., Alamri A., Ahmad F., Krishnaraju V. (2022). The Unique Carboxymethyl Fenugreek Gum Gel Loaded Itraconazole Self-Emulsifying Nanovesicles for Topical Onychomycosis Treatment. Polymers.

[48] Shah V.H., Jobanputra A. (2018). Enhanced Ungual Permeation of Terbinafine HCl Delivered Through Liposome-Loaded Nail Lacquer Formulation Optimized by QbD Approach. AAPS PharmSci. Tech..

[49] Kushwaha A.S., Repka M.A., Narasimha Murthy S. (2017). A Novel Apremilast Nail Lacquer Formulation for the Treatment of Nail Psoriasis. AAPS PharmSci. Tech..

[50] Murthy S.N., Vaka S.R.K., Sammeta S.M., Nair A.B. (2009). TranScreen-N™: Method for rapid screening of trans-ungual drug delivery enhancers. J. Pharm. Sci..

[51] Aggarwal R., Targhotra M., Sahoo P.K., Chauhan M.K. (2020). Efinaconazole nail lacquer for the transungual drug delivery: Formulation, optimization, characterization and in vitro evaluation. J. Drug Deliv. Sci. Technol..

[52] Rahman A., Aqil M., Ahad A., Imam S.S., Qadir A., Ali A. (2021). Application of central composite design for the optimization of itraconazole loaded nail lacquer formulation. 3 Biotech.

[53] Joshi M., Sharma V., Pathak K. (2015). Matrix based system of isotretinoin as nail lacquer to enhance transungal delivery across human nail plate. Int. J. Pharm..

[54] Brown M.B., Khengar R.H., Turner R.B., Forbes B., Traynor M.J., Evans C.R.G., Jones S.A. (2009). Overcoming the nail barrier: A systematic investigation of ungual chemical penetration enhancement. Int. J. Pharm..

[55] Krawczyk-Santos A.P., da Rocha P.B.R., Kloppel L.L., Souza B.d.S., Anjos J.L.V., Alonso A., de Faria D.L.A., Gil O.M., Gratieri T., Marreto R.N. (2021). Enhanced nail delivery of voriconazole-loaded nanomicelles by thioglycolic acid pretreatment: A study of protein dynamics and disulfide bond rupture. Int. J. Pharm..

[56] Shivakumar H., Vaka S.R.K., Madhav N.S., Chandra H., Murthy S.N. (2010). Bilayered nail lacquer of terbinafine hydrochloride for treatment of onychomycosis. J. Pharm. Sci..

[57] Saner M.V., Kulkarni A.D., Pardeshi C.V. (2014). Insights into drug delivery across the nail plate barrier. J. Drug Target..

